# Assessment of Smartphone Apps for Common Neurologic Conditions (Headache, Insomnia, and Pain): Cross-sectional Study

**DOI:** 10.2196/36761

**Published:** 2022-06-21

**Authors:** Mia T Minen, Alexis George, Erica Camacho, Leslie Yao, Ananya Sahu, Maya Campbell, Mia Soviero, Quazi Hossain, Deepti Verma, John Torous

**Affiliations:** 1 Department of Neurology New York University Langone Health New York, NY United States; 2 Department of Psychiatry Beth Israel Deaconess Medical Center New York, NY United States; 3 Barnard College New York, NY United States; 4 The City College of New York New York, NY United States

**Keywords:** headache, pain, insomnia, mobile health, smartphone apps, mobile phone

## Abstract

**Background:**

There are thousands of apps for individuals struggling with headache, insomnia, and pain, but it is difficult to establish which of these apps are best suited for patients’ specific needs. If clinicians were to have access to a platform that would allow them to make an informed decision on the efficacy and feasibility of smartphone apps for patient care, they would feel confident in prescribing specific apps.

**Objective:**

We sought to evaluate the quality of apps for some of the top common, disabling neurologic conditions (headache, insomnia, and pain) based on principles derived from the American Psychiatric Association’s (APA) app evaluation model.

**Methods:**

We used the Mobile Health Index and Navigation database and expanded upon the database’s current supported conditions by adding 177 new app entries. Each app was rated for consistency with the APA’s app evaluation model, which includes 105 objective questions based on the following 5 major classes of consideration: (1) accessibility, (2) privacy and security, (3) clinical foundation, (4) engagement style, and (5) interoperability. These characteristics were evaluated to gain a broader understanding of the significant features of each app category in comparison against a control group.

**Results:**

Approximately 90% (187/201) of all apps evaluated were free to download, but only 50% (63/201) of headache- and pain-related apps were truly free. Most (87/106, 81%) sleep apps were not truly free to use. The apps had similar limitations with limited privacy, accessibility, and crisis management resources. For example, only 17% (35/201) of the apps were available in Spanish. The apps offered mostly self-help tools with little tailoring; symptom tracking was the most common feature in headache- (32/48, 67%) and pain-related apps (21/47, 45%), whereas mindfulness was the most common feature in sleep-related apps (73/106, 69%).

**Conclusions:**

Although there are many apps for headache, pain, and insomnia, all 3 types of apps have room for improvement around accessibility and privacy. Pain and headache apps share many common features, whereas insomnia apps offer mostly mindfulness-based resources. Given the many available apps to pick from, clinicians and patients should seek apps that offer the highest-quality features, such as complete privacy, remedial features, and the ability to download the app at no cost. These results suggest that there are many opportunities for the improvement of apps centered on headache, insomnia, and pain.

## Introduction

There is a health crisis in the United States whereby people cannot access neurologic care in a timely manner [1]. As smartphones and digital tools increase in popularity, with 85% ownership as of 2021, compared to only 35% in 2011 [2], many mobile health tools have been developed as a means to provide self-management and other strategies to patients. This is especially true for common and disabling neurologic conditions such as headache, sleep, and pain disorders [3]. With 1 in every 6 American adults experiencing migraine and severe headache [4], 70 million American adults experiencing sleep problems [5], and 1 in 5 American adults experiencing chronic pain [6], there is a clear need for treatment. Although a quick search in an app store for “headache,” “pain,” or “sleep” may reveal countless apps, the apps listed at the top of a search result do not necessarily offer benefits in terms of utility and efficacy compared to others [7]. There have been many reviews of mental health apps in each of the app store marketplaces (ie, Apple iTunes and Google Play) [8], but there have been fewer reviews for neurology-focused apps [9]. Given the inherent risks of apps, including privacy concerns [10-12], mixed evidence around efficacy, and broad usability concerns [13,14], clinicians and patients need to be aware of the state of these public-facing apps and be able to understand their risks and benefits.

Despite the broad risks in the digital health space, emerging evidence suggests the potential benefits of apps for neurological conditions. Even simple headache tracking apps have been shown to help with the management of symptoms [15]. In randomized controlled trials, apps for insomnia have shown benefits such as significant reduction in sleep-related impairment of quality of life and mental well-being [16]. Presently, apps for pain management are expanding in scope, with features such as pain impact recording and medication tracking [17]. To aid users in their efforts to discover apps that are accessible, safe, effective, and evidence based, several app libraries have been developed. One such publicly available tool that considers these and many other metrics when evaluating an app is the Mobile Health Index and Navigation Database (MIND) [8,18]. MIND is the largest open and publicly accessible database of mental health and neurology-focused apps—with over 600 apps, each rated across 105 criteria and updated at least every 6 months. Per recent research, MIND is unique as it also represents diversity, equity, and inclusion criteria, such as accessibility features and language options, which offer a more comprehensive window into apps utility [19]. To understand the current state of mobile apps for neurological disorders, using headache and pain as the leading causes of nonfatal health loss [20] and insomnia as the common sleep disorder [21], we applied the 105 metrics found in MIND to the top neurological apps discoverable on iOS and Android devices.

We sought to (1) search app stores for headache-, pain-, and sleep-related apps and review them using the MIND database, and (2) evaluate the characteristics of these apps and compare them to a control group of apps across unique features not yet reported in the literature, including language (Spanish), crisis management, and the ability to connect with providers. We predict that if clinicians were to have access to a platform that would allow them to make an informed decision on the efficacy and feasibility of smartphone apps for patient care, they would feel confident in prescribing specific apps.

## Methods

### App Selection

**Figure 1:**
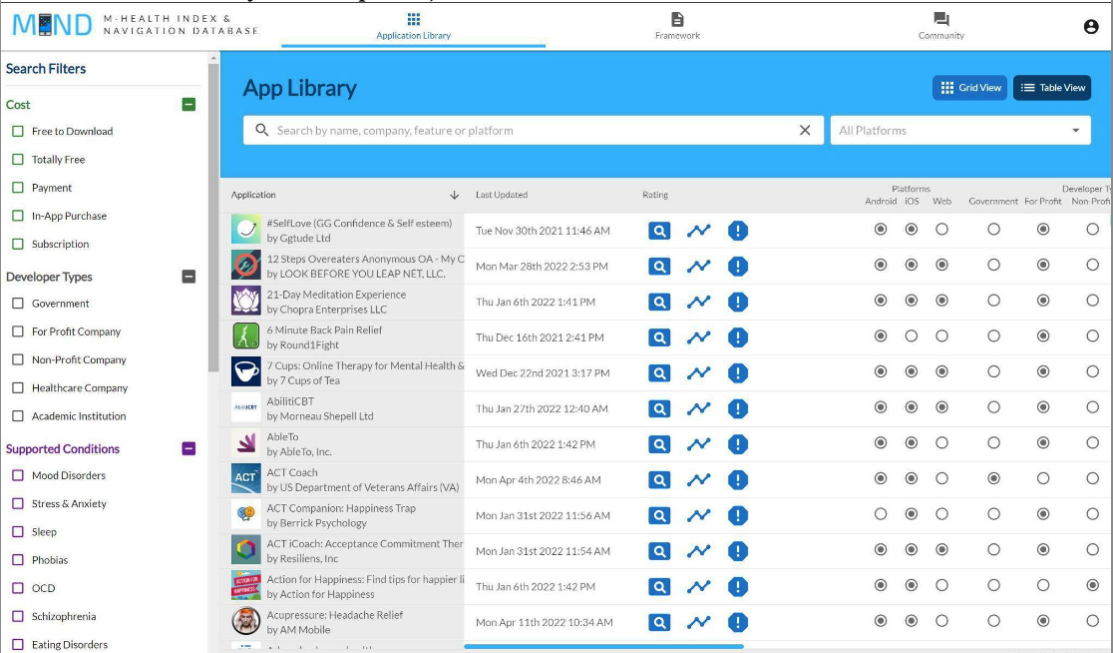
Screenshot of the main page of the MINDapps database taken in April 2022 (the screenshot was taken after the study was completed).

This study used the MIND database, published by the Division of Digital Psychiatry at Beth Israel Deaconess Medical Center [18]. Details about MIND have been published previously [8,22,23]. In brief, MIND is the largest publicly available database of mental health apps; it currently logs 656 mental health apps in the commercial market across a variety of supported conditions and acts as an open resource for users to filter apps by features of their personal preference. To add an app to the MIND database, there are 105 objective questions that are answered based on the following 5 major classes of consideration within the American Psychiatric Association’s app evaluation model: accessibility, privacy and security, clinical foundation, engagement style, and interoperability (Multimedia Appendix 1). All data in the MIND database are publicly accessible through the MINDapps website [18]. A screenshot of the MIND database is provided in Figure 1.

**Figure 2:**
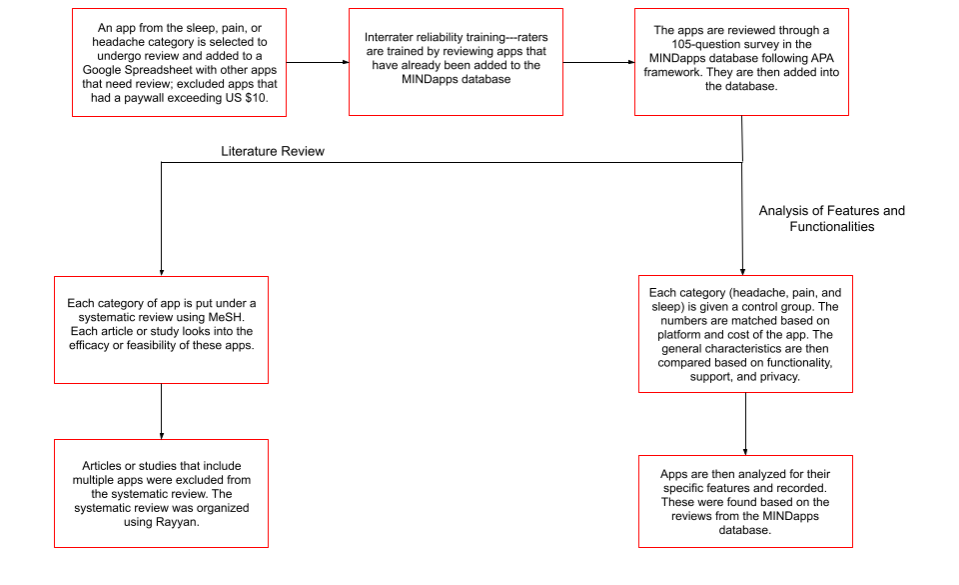
Flowchart detailing app selection and app rating process. APA: American Psychiatric Association; MeSH: Medical Subject Headings.

The database does not include apps for which the cost of download exceeds US $10 or those not accessible to the public. Prior to this study, the database had 24 apps for pain-, headache-, or sleep-related conditions. The study expanded upon the current database by adding 177 new app entries for the supported conditions—47 apps for pain, 48 for headache, and 106 for sleep—accounting for overlap between categories, amounting to a total of 201 apps analyzed in this study. The selection of new apps was conducted as follows: to gain an understanding of the app marketplace for headache-, pain-, and sleep-related apps, terms such as “headache,” “pain,” and “insomnia” were searched in both the iOS and Google Play stores in June and July 2021. The first 50 apps that appeared on each platform (Apple App Store and Google Play Store) for each search term (headache, pain, and sleep) were compiled into a Google Sheet. Thus, 300 apps were discovered in our preliminary search. Some of these apps appeared within the first 50 apps searched on both platforms. Apps were assessed for relevance to the neurological conditions of interest. Given that there is no standard definition for these apps and many apps appearing in a search may not be related to wellness or health (eg, a gaming app), all apps selected for evaluation were agreed upon for relevance via consensus of all raters, all of whom are authors. Apps were excluded if they were irrelevant, clinician facing, nonfunctional, unavailable in English, and required an access code to use them. Upon removal of apps that did not meet the inclusion criteria, the remaining 177 relevant apps were evaluated and added to the MIND database. Figure 2 shows a flowchart detailing app selection and app rating process.

Given that all data were entered into the MIND, unique to this study, we sought to publicly share all app evaluations, so others can expand on these results and use this raw data to explore and find relevant apps today, as well as to aid neurologists in making informed decisions around choosing smartphone apps for patients with these conditions.

### Statistical Analysis

A total of 6 app raters underwent interrater reliability training [23], and 1 app rater evaluated each app. Interrater reliability was assessed using Cohen κ statistic, for which raters demonstrated very good interrater reliability (defined as a κ value above 0.750), with an average κ value of 0.859 across all apps rated. Discrepancies between the raters were addressed individually through discussion and subsequently resolved by clarifying any discrepancy in the description of each question.

As this was purely an exploratory study, we used Excel (Microsoft Corporation) and reported descriptive statistics.

## Results

### Overview

**Figure 3:**
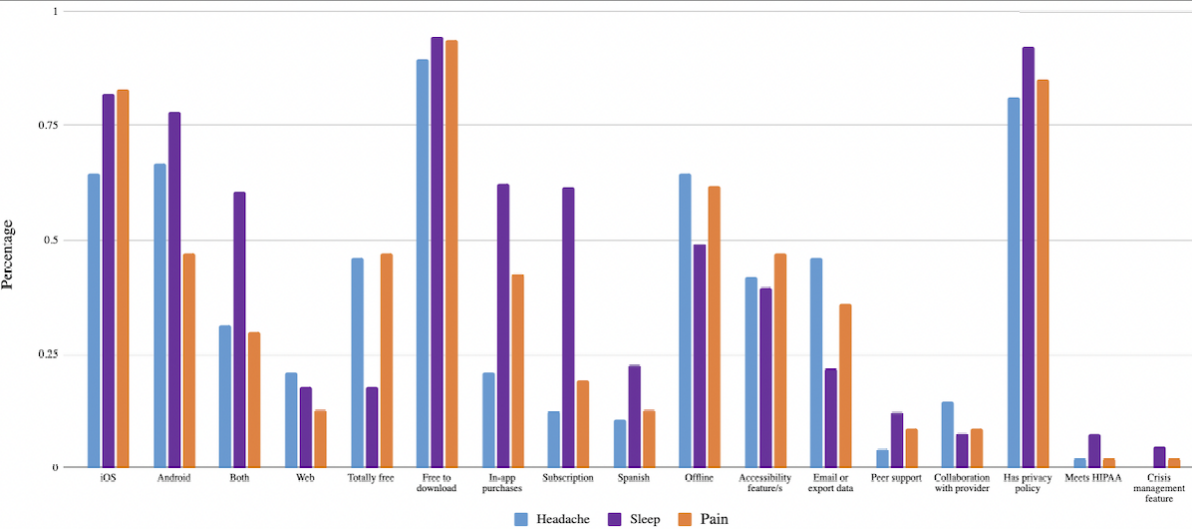
Characteristics and features of headache, pain, and sleep apps. HIPPA: Health Insurance Portability and Accountability Act.

Of the 656 apps available in MIND in July 2021, a total of 201 were related to headache, pain, or insomnia. Overall, these 201 apps focusing on headache, pain, and insomnia offered common features for tracking symptoms, tracking medication, journaling, and psychoeducation. Very few apps (26/201, 13%) used biological data, defined as metrics obtained from external devices (eg, wearables or built-in phone sensors), to monitor personal health. Examples of biodata collected include skin conductance, heart rate, and sleep quality. Across all 3 types of apps examined, we found similarities in terms of platform cost, special features (eg, Spanish language and accessibility features), clinician support, and privacy features as shown in both Table 1 and Figure 3.

**Table 1:** General characteristics of headache and migraine apps, sleep and insomnia apps, and pain-related apps (N=201).

Characteristics		Apps, n (%)		
		Headache and migraine (n=48)	Sleep and insomnia (n=106)	Chronic pain (n=47)
**Platforms**				
	iOS	31 (65)	87 (82)	39 (83)
	Android	32 (65)	83 (78)	22 (47)
	Both iOS and Android	15 (31)	64 (60)	14 (30)
	Web	10 (21)	19 (18)	6 (13)
**Cost**				
	Totally free	22 (46)	19 (18)	22 (47)
	Free to download	43 (90)	100 (94)	44 (94)
	In-app purchases	17 (21)	66 (62)	20 (43)
	Subscription	10 (13)	65 (61)	9 (19)
**Functionality**				
	Spanish	5 (11)	24 (23)	6 (13)
	Offline	31 (65)	52 (49)	29 (62)
	Accessibility features	20 (42)	42 (40)	22 (47)
	Email or export data	25 (46)	23 (22)	17 (36)
**Support**				
	Peer support	2 (4)	13 (12)	4 (9)
	Collaboration with provider	7 (15)	8 (8)	4 (9)
**Privacy**				
	Includes privacy policy	39 (8)	98 (92)	40 (85)
	Meets HIPAA^a^ requirements	1 (2)	5 (5)	1 (2)
	Crisis management feature	0 (0)	8 (8)	1 (2)

^a^HIPAA: Health Insurance Portability and Accountability Act.

### Accessibility

Apps were accessible on Apple, Android, and web browsers, although less than 50% (63/201) were truly free of cost. Over 90% (187/201) of apps in all categories were free to download, but this did not guarantee no-cost or even low-cost use. Over 50% (112/201) of apps across all disease states offered functionalities for working offline, that is, without an internet connection. Approximately 17% (35/201) of these apps supported Spanish.

### Crisis Management and Privacy

Apps in all 3 categories demonstrated lack of crisis management features, with 0% (0/48) of headache-related apps, 2% (1/47) of pain-related apps, and 8% (8/106) of sleep-related apps offering crisis resources in terms of providing resources for a hotline or contact with a medical professional. Most apps for pain, headache, or insomnia did offer a privacy policy, with 88% (177/201) of apps among all categories containing information on user data storage and usage. Although apps that are not part of health care accountability organizations are not subject to the Health Insurance Portability and Accountability Act (HIPAA), 3% (7/201) of apps stated that they were HIPAA compliant.

### Self-help and Hybrid Use With a Clinician

Most apps were self-help centered, but some included collaboration of a clinician (either from the app or outside the app). This was offered by 15% (7/46) of headache apps, 9% (8/47) of pain apps, and 8% (4/106) of sleep apps. Proportions of apps offering peer support were similarly low.

### Overall Functionality

The reported functionality offered by these apps is shown in Table 2. Results show that headache and pain apps shared many common features, with tracking symptoms as the most used feature and mindfulness as one of the least used features. In contrast, the apps that focused on sleep had mindfulness as their most common feature and symptom tracking as one of the least common features.

**Table 2:** Top features for headache and migraine apps, sleep and insomnia apps, and pain-related apps (N=201).

Apps and features			Values, n (%)
**Headache and migraine apps (n=48)**			
	Track symptoms	32 (67)	
	Track medication	30 (63)	
	Journaling	16 (33)	
	Psychoeducation	14 (29)	
	Track sleep	6 (13)	
	Track mood	6 (13)	
	Physical health	5 (11)	
	Mindfulness	5 (11)	
	Biodata	5 (11)	
**Sleep and insomnia apps (n=106)**			
	Mindfulness	73 (69)	
	Deep breathing	58 (55)	
	Psychoeducation	32 (30)	
	iCBT^a^ or sleep therapy	26 (25)	
	Track mood	26 (25)	
	Goal settings or habits	25 (24)	
	Journaling	25 (24)	
	Track sleep	24 (23)	
	Physical health	15 (14)	
**Pain apps (n=47)**			
	Track symptoms	21 (45)	
	Physical health	20 (43)	
	Track medication	16 (34)	
	Psychoeducation	15 (32)	
	Physical health exercises	12 (26)	
	Journaling	10 (21)	
	Track sleep	8 (17)	
	Track mood	8 (17)	
	Mindfulness	6 (13)	

^a^iCBT: internet-based cognitive behavioral therapy.

### Other Considerations

Each app was evaluated across 105 individual criteria, and all results are publicly accessible and searchable today through the MINDapps database [18]. Figure 1 shows an example of how a reader can interactively explore and search these apps across individual questions. We do not provide scores for certain categories (privacy, functionality, etc), as MINDapps allows users to select their own filters and create their own criteria based on personal needs.

## Discussion

### Principal Findings

Our review of apps for headache, insomnia, and pain is the largest review of publicly available neurology-focused offerings to date, with results derived from over 200 apps each categorized across 105 dimensions. Pain and headache apps share many common features, whereas insomnia apps offer mostly mindfulness-based resources. We found that apps mostly offered self-help tools with little tailoring, and that symptom tracking was the most common feature in headache- (32/48, 67%) and pain-related apps (21/47, 45%), whereas mindfulness was the most common feature in sleep-related apps (73/106, 69%). Despite the number of apps and 3 unique conditions, we found numerous commonalities, including limited privacy, accessibility, and crisis management resources, in these mostly self-help tools. In terms of features offered, tracking and mindfulness-related features were most common, with individual apps offering varied ratios or types of these core features. These results suggest opportunities for innovation around the structure of apps themselves, as well as how they deliver tracking or mindfulness, with any innovation presenting transdiagnostic benefits. The numerous overlapping features offered by these apps also suggest that clinicians and patients today can be demanding in selecting an app, as there are likely minimal differences in their core functionality. Using MINDapps [18], they can explore which apps may offer the exact app features desired.

In selecting apps beyond core functions, our results highlight concerns about the structure of apps in terms of privacy, accessibility, and use. Although it is well known that most health-related apps had privacy and access issues, our results are novel for apps in the neurology field. A June 2021 review of 20,911 Android apps across the entire digital health space found that 28.1% of apps offer no privacy policy [24], and our results showed this to be the case in only 9% (177/201). This lower proportion is encouraging but may also be due to only including apps that appeared to be clinically relevant. On September 15, 2021, the Federal Trade Commission (FTC) noted that for wellness apps not covered by HIPAA, the FTC will now expect them to follow HIPAA-related rules around breaches, suggesting that apps will need to offer a change in the required security process [25].

A recent review of mental health–focused apps found that almost 15% supported Spanish, which is consistent with our result of 17% (35/201) for the neurology-focused apps we reviewed [26]. This result suggests an immediate opportunity to increase reach, while better supporting diversity and inclusion. Most apps we reviewed were self-help focused, with only a small fraction designed to be used in partnership with a peer or clinicians. Across the broader digital health field, there is growing evidence that apps used in partnership with others may be more engaging and effective than the self-help ones. Lessons already learned about low engagement with mental health apps [27] may help these neurology-related apps develop as more engaging relationship-based tools that could offer more support for hybrid use.

### Comparison With Prior Work

Our findings about the common features underlying apps for headache, insomnia, and pain suggest room for transdiagnostic innovation around all these apps. The common features of symptom tracking, and mindfulness are also the most common features in mental health apps [8], suggesting a potential synergy between these two fields. This result makes sense as apps offer a practical platform to deliver behavioral-based treatments and remotely capture symptoms, which are themselves shared aspects between psychiatry and neurology. Although the insomnia apps focused more on behavioral interventions, the headache and pain apps focused more on symptom tracking.

Furthermore, although smartphone apps may show promising results for patient care, it remains challenging to evaluate their effectiveness. Clinical studies do not often feature a valid control condition or simulate the challenges of real-world app engagement, which is frequently low [27]. Thus, the MIND framework does not rate the quality of scientific evidence for apps nor their engagement, given the lack of consensus or data availability around these points. Our results are therefore best interpreted as signals of what these apps are claiming to offer, with the recommendations for personal use and exploration with the app itself to determine whether the feature meets the needs for each clinical use case. Clinicians should feel empowered to go to MINDapps [18] and search for apps that they feel would meet the standards for their patient’s needs. The results of this paper can help calibrate expectations and guide clinicians in searching for apps that meet the unique demands of each patient served.

### Limitations

Although each app was downloaded and tested, some aspects of the data coded within this study are based on the description of the app itself, such as who the developer is and what the privacy policy reports. As a result, coded information could be inaccurate. Although app descriptions may hold biases, an advantage of this approach is that apps are constantly being updated, and it is feasible and practical to update apps on the website regularly. Apps were reviewed by only 1 rater. It was also found that there were several apps that falsely advertised their features; other times, the majority of the features were locked behind a paywall, so they could not be seen or used unless the premium version was paid for. Although this study represents perhaps the largest analysis of neurology-related apps, there remains no simple way to identify all relevant apps, given the limitations in searching function on the Apple and Android marketplaces.

### Conclusion

Although the number of headache, sleep, and pain apps on the market continues to expand, there are numerous opportunities for content improvement. Many of these apps were lacking in privacy, accessibility, and crisis management resources—features that would significantly improve the app platforms. The results of this study suggest an opportunity for improvement in app structure and the delivery of important features. Patient care may be improved with the incorporation of a transdiagnostic approach to health-based smartphone apps.

## Appendix

Multimedia Appendix 1 App review questions.

## Abbreviations

FTC: Federal Trade Commission
HIPAA: Health Insurance Portability and Accountability Act
MIND: Mobile Health Index and Navigation
